# Development of a food frequency questionnaire for Sri Lankan adults

**DOI:** 10.1186/1475-2891-11-63

**Published:** 2012-08-31

**Authors:** Ranil Jayawardena, Sumathi Swaminathan, Nuala M Byrne, Mario J Soares, Prasad Katulanda, Andrew P Hills

**Affiliations:** 1 Institute of Health and Biomedical Innovation, Faculty of Health, Queensland University of Technology, Brisbane, Queensland, Australia; 2 Diabetes Research Unit, Department of Clinical Medicine, Faculty of Medicine, University of Colombo, Colombo, Sri Lanka; 3 St John's Research Institute, St John’s National Academy of Health Sciences, Bangalore, India; 4 Curtin Health Innovation Research Institute, School of Public Health, Curtin University, Perth, Western Australia; 5 Mater Mother’s Hospital, Mater Medical Research Institute and Griffith Health Institute, Griffith University, Brisbane, Queensland, Australia

**Keywords:** Food frequency questionnaire, Development, FFQ, Sri Lanka, Adults

## Abstract

**Background:**

Food Frequency Questionnaires (FFQs) are commonly used in epidemiologic studies to assess long-term nutritional exposure. Because of wide variations in dietary habits in different countries, a FFQ must be developed to suit the specific population. Sri Lanka is undergoing nutritional transition and diet-related chronic diseases are emerging as an important health problem. Currently, no FFQ has been developed for Sri Lankan adults. In this study, we developed a FFQ to assess the regular dietary intake of Sri Lankan adults.

**Methods:**

A nationally representative sample of 600 adults was selected by a multi-stage random cluster sampling technique and dietary intake was assessed by random 24-h dietary recall. Nutrient analysis of the FFQ required the selection of foods, development of recipes and application of these to cooked foods to develop a nutrient database. We constructed a comprehensive food list with the units of measurement. A stepwise regression method was used to identify foods contributing to a cumulative 90% of variance to total energy and macronutrients. In addition, a series of photographs were included.

**Results:**

We obtained dietary data from 482 participants and 312 different food items were recorded. Nutritionists grouped similar food items which resulted in a total of 178 items. After performing step-wise multiple regression, 93 foods explained 90% of the variance for total energy intake, carbohydrates, protein, total fat and dietary fibre. Finally, 90 food items and 12 photographs were selected.

**Conclusion:**

We developed a FFQ and the related nutrient composition database for Sri Lankan adults. Culturally specific dietary tools are central to capturing the role of diet in risk for chronic disease in Sri Lanka. The next step will involve the verification of FFQ reproducibility and validity.

## Background

It is widely recognized that an unhealthy diet is a major risk factor for many of the chronic non-communicable diseases and improving dietary habits is not simply an individual but a societal problem [1]. However, it is difficult to assess the dietary habits of free-living individuals because of variability in food preference and availability, socio-economic factors, cultural concerns and educational level [2]. National dietary surveys have several important functions and provide valuable information on dietary habits and nutritional status. Moreover, nutritional monitoring is important for implementation of programs related to food, nutrition, and health promotion for any country serious about promoting the health and wellbeing of its population [3]. Food Frequency Questionnaires (FFQs) are the most common dietary assessment tool used in large epidemiologic studies of diet and health [4]. To cater for differences in food based on cultural and regional factors, numerous FFQs have been developed comprising the list of foods commonly eaten in a particular country or by a particular population.

Sri Lanka is a ‘low-middle’ income country in South Asia with a population of nearly 21 million [5]. Sinhalese is the main ethnic group but there are significant proportions of Tamils and Moors living in different parts of the country. With recent economic development, urbanization and changes in lifestyle patterns, Sri Lanka is experiencing a nutritional transition with the coexistence of under-nutrition and overweight and obesity [6,7]. A high prevalence of iron deficiency anemia among pregnant women, and subclinical vitamin A deficiency, stunting and wasting among pre-school children, are still major public health problems [8]. Recent studies in Sri Lanka indicate a high prevalence of diabetes mellitus with one in every five adults aged above 20 years having either diabetes or pre-diabetes [9], and the prevalence of hypertension, obesity, dyslipidaemia in urban areas are reaching epidemic proportions [10,11]. A quarter of adults is suffering from metabolic syndrome [12]. In the Sri Lankan context, diet-related chronic diseases currently account for an estimated 18.3% of total mortality and 16.7% of hospital expenditure [7].

Despite strong indications of a rise in lifestyle-related non-communicable diseases (NCDs) in Sri Lanka published guidelines are not justified with sound research evidence on dietary habits [13]. There is a paucity of data on the dietary habits of Sri Lankans and in order to assess dietary intake, a culturally specific dietary assessment tool is necessary. This paper describes the development of a FFQ for Sri Lankan adults designed to assess and monitor dietary intake and be used to assist in national level programs to combat non-communicable diseases.

## Methods

### Study sample

Data were collected from a subset of the national ‘Sri Lanka Diabetes and Cardiovascular Study’ (SLDCS) using a multi-stage, stratified, random sampling procedure (n = 500) [9]. However, data collection in the SLDCS was affected by the prevailing civil war which resulted in no data being collected from Northern and Eastern provinces. To obtain a nationally representative sample, additional subjects (n = 100) were later recruited from the two provinces using similar selection criteria. Details of subject selection are published elsewhere [14].

### Data collection

Selected households were contacted via telephone and the purpose of the study explained and verbal consent taken. Where telephone facilities or contact phone numbers were unavailable, households were visited by the study team with prior postal notice. Subsequently, households were visited on a random day and dietary and demographic details obtained after informed written consent was provided. An interviewer-administered questionnaire was used for data collection and information regarding socio-demographic factors and 24-h dietary recall (24DR) was obtained. Two trained nutritionists retrieved dietary data using a standardized manual of procedure. Food portion sizes were obtained from participants using standard household measures such as a plate, bowl, cup, glass, and spoons of different size; as well as using photographs (Additional file 1) of food portion size and a food atlas [15,16].

### Development of a nutrient composition database

It is essential to have food composition values to convert information from an FFQ into macronutrient and micronutrient values. Currently the database on Sri Lankan dishes is meagre. We therefore compiled information from the food composition tables of Sri Lanka [5], United States Department of Agriculture nutrient database (USDA) [17], the Indian Food Composition Tables [18], and McCance and Widdowson’s food composition tables [19] to develop a comprehensive and new nutrient composition database as follows:

a) Nutrition values for single food items were taken mainly from the USDA nutrient database.

b) Nutrition information leaflets or details from direct contact with producers were used for locally available food products (e.g. biscuits).

c) For mixed dishes and cooked foods, local recipes were taken from popular cookery books [20] and by interviewing participants. All recipes were accepted after checking for face validity by consulting local housewives and nutritionists. According to the recipes, ingredients were weighed to the nearest 1 g for edible portions of the foods, and the food items were cooked and weighed. Nutritional composition of the final recipe was calculated by entering nutritional values and weights of individual ingredients into a spreadsheet. The sum of each nutrient was computed and standardised to 100 g of the final product. Data on weight loss associated with cooking (e.g. due to water evaporation) was recorded to ensure accurate nutrient density of the portion size consumed. However, nutrient losses (e.g. vitamins) during food preparation were not considered.

Newly developed food composition data for each recipe was entered into NutriSurvey 2007 (EBISpro, Germany) nutrient analysis software.

### Development of the FFQ

In addition to 24-h recalls, a number of additional methods were used to obtain a more comprehensive food list.

Open ended questions were asked to capture details of seasonal fruits and festival foods.

Alcohol intake and use of dietary supplements were collected separately.

Local nutrition experts were contacted to obtain unreported foods for the different ethnic groups.

Food items were divided into eight groups by two independent nutritionists and included: 1) cereals or equivalents; 2) vegetables; 3) pulses; 4) meat or equivalents; 5) fruits; 6) drinks; 7) miscellaneous; and 8) alcohol.

Using stepwise multiple regression analysis, food items that contributed to a cumulative 90% of the variance in energy, carbohydrates, fat, protein and dietary fibre were included in the FFQ.

Food items with similar consumption patterns and nutrients were aggregated into groups on the basis of their energy, carbohydrate, fat, protein and dietary fibre.

To improve the quantification of food intake we included food photographs to estimate habitual portion sizes for those foods that could not be easily assessed in natural units or household measures. The FFQ contains colour photographs of 3 different sized portions of four commonly consumed foods namely rice, vegetable, chicken, and dhal (lentil). For each food, photograph A represents the 25^th^ percentile, B the median and C the 75^th^ percentile of the distribution of serving sizes reported in this study. Seven different serving sizes can be attributed to each food class, by selecting serving sizes that are equal to A, B or C, less than A, greater than C and between A-B or between B-C [21]. However, we found low clarity in determining portion sizes from pre-test subjects when vegetables, meat and dhal were pictured alone on the plate. In Sri Lanka, curries are served on rice, not as a side dish; therefore, to improve precision and accuracy regarding portion sizes, we displayed individual food items on the medium rice plate (B) as the background.

The majority of FFQs from developed countries have used several frequency categories [22,23]. However, this is not the case in Southern India [24]. To some extent, open-ended response scales (number of units taken at a time: ‘per day’, ‘week’, ‘month’ or ‘year’) reflect the precision with which participants can realistically describe their usual intake. We also used an open-ended response scale. FFQs were designed to incorporate interviewer-administered methods. A protocol was developed to obtain data uniformly. The questionnaire was pilot tested for clarity, interpretation and improvement of format in 25 individuals who had similar demographic characteristics to the study group but who were not participants in the study. All statistical analyses were undertaken using SPSS version 16 (SPSS Inc., Chicago, IL, USA) and independent sample *T*-test was used to compare demographic characteristics and nutrient intake between men and women. The significance level was set at 0.05 in all analyses.

## Results and Discussion

From the total sample of 600, 482 completed (Male = 166; Female = 316) all demographic, anthropometric and dietary profiles. The demographic profile of the study population is shown in Table 1. Overall, there was a preponderance of Sinhalese followed by Tamils and Moors. Males had lower BMI values compared to their female counterparts (M: 22.0 ± 3.5 *vs.* 23.7 ± 4.3 kg/m^2^; p < 0.05). Average daily energy intake was 1656.7 ±535.0 kcal, with significantly higher caloric consumption by men compared to women (p < 0.05). The main source of energy was from carbohydrates for both men and women. Total protein and fat intake for men was 52.8 ±43.0 g/day and 40.5 ±18.1 g/day respectively and for women, 40.0 ±13.9 g/day and 31.9 ±14.1 g/day (Table 2).

**Table 1:** Demographic characteristics of the sample

**Variables**	**Males (169)**	**Female (321)**
Age (y)	48.4 ± 15.6	48.1 ± 14.1
BMI (kg/m^2^)*	22.0 ± 3.5	23.7 ± 4.3
Area of Residence		
·Urban	27.8 (47)	36.1 (116)
·Rural	60.4 (102)	57.6 (185)
·Estate	11.8 (20)	6.2 (20)
Ethnicity (%)		
·Sinhalese	71.0 (120)	80.1(257)
·Moors	4.7 (8)	7.2(23)
·Sri Lankan Tamil	11.8 (20)	7.2(23)
·Indian Tamil	12.4 (21)	5.6(18)
Education level (%)		
·No Schooling	6.5 (11)	6.5 (21)
·Upto 5 years	27.2 (46)	25.2(81)
·Upto 11 years	34.9(59)	40.5(130)
·Upto 13 years	27.2(46)	22.7(73)
·Graduate	4.1(7)	5.0(16)
		

Data are mean ± SD. Values in parenthesis are total number. *p < 0.05.

**Table 2:** Nutrient intake of the study population

**Characteristics**	**Total e (SD)**	**Male**	**Female**
Energy	1656.7 (535)	1912.7(566.9)*	1513.6 (458.5)
Carbohydrates	304.4(103.1)	352.4 (110.3)*	277.5 (88.3)
Protein	44.6 (28.8)	52.8 (43)*	40 (13.9)
Total fat	35.0 (16.1)	40.5 (18.1)*	31.9(14.1)
Dietary fibre	18.1(8.4)	21.3(9.2)	16.3(7.3)

Values are mean ± SD. *p < 0.05.

In this study 312 different food items were recorded. Nutritionists grouped similar food items which resulted in a total of 178 food items. After performing step-wise multiple regression, 93 foods explained 90% of the variance for total energy intake, carbohydrates, protein, total fat and dietary fibre. Subsequently, conceptually similar food items were grouped together yielding a final list of 81 food items (Table 3). An additional nine food items were included to cover festival and seasonal dietary habits and the final 90 food items were categorized as cereal or equivalents (n = 19), vegetables (n = 20), pulses (n = 6), meat or alternatives (n = 10), fruits (n = 9), beverages (n = 7), miscellaneous (n = 14), and alcohol (n = 5).

**Table 3:** Elements of the food frequency questionnaire

	**Cereals or equivalents**	**Vegetables**	**Pulses**	**Meat or alternatives**	**Fruits**	**Beverages**	Miscellaneous*	**Alcohol**
Total food items and mixed dishes	36	48	11	17	19	13	29	2
Contribution of 90%	28	21	5	9	9	7	12	2
Grouping of food items	19	18	5	9	9	7	12	2
Inclusion of foods	0	2	1	1	1	0	2	3
Final food items	19	20	6	10	9	7	14	5

* Dairy, sweets, desserts, nuts.

The paper describes the process of development of a FFQ for Sri Lankan adults using a nationally representative sample. Dietary assessment of this population is invaluable to understand the role of nutrition in chronic disease so that preventive strategies can be implemented. The aim of dietary assessment of populations is to rank people by a measure of usual rather than current diet. The strengths of this study include a nationally representative sample of Sri Lankan adults and the creation of a comprehensive new database for nutrient analysis. However, males are under-represented in this study which stems from data collection being on a random day when most males were engaged in active occupations away from home. However, in Sri Lanka family members consume similar foods; therefore, obtaining dietary data from females did not significantly affect the food list in our study. The number of food items in a FFQ is a crucial factor in determining the accuracy of the data and the practicability of the questionnaire. Many FFQs have between 100–150 items [25] and the risk of over-reporting through increased subject burden increases with the large number of items [22,25]. In our FFQ, we have 90 items and 12 photos of food items to enable an accurate estimation of dietary exposure.

Sri Lanka as a tropical island has no clear four seasons but two monsoons influence cultivation. Hence additional seasonal fruits and vegetables are also included in our FFQ. Over 55% of adult males are current alcohol drinkers [26]; however in our data collection alcohol consumption was under-reported (0.5% of participants) with no women reporting the consumption of alcohol. In Sri Lanka, drinking alcohol has negative social and religious stigma. Thus, common alcoholic beverages were added to the FFQ. Dietary recalls indicated differences between the ethnic groups in the type of nutrients derived from different food sources. The main carbohydrate source varied among ethnic groups; Indian Tamils reported consuming wheat flower (as Roti) whereas Sinhalese eat rice as the main staple food and Sri Lankan Tamils consume Dose, Itale and Wade frequently. Ethnicity was an important factor in the selection of foods containing protein, not surprisingly; pork and beef consumption was not reported by Moors (Muslims) and Tamils, respectively.

A variety of methods are available to collect food consumption data but a common challenge for individual-based dietary assessment methods is portion size estimation. Although weighing served portions is often considered the gold standard; for practical reasons, portion estimation using photographs are used among both adults and children [27]. A study conducted in Burkina Faso showed that food photographs are valuable for the quantification of food portion size among rural and less educated middle-aged women [28]. Men usually consume larger portions than women [29] and the use of photographs helps to categorize gender variation in portion sizes more precisely. This is crucial to obtaining reliable estimates of macronutrient and micronutrient intakes. Several countries use FFQs with photo series and scoring systems [30,31].

The main weakness of the previous national level NCD survey (SLDCS) was the absence of nutritional data on the population and their relationship with the high NCD risk in the country. One of the main objectives of the current work was to develop a FFQ to administer in the next national level NCD survey. Moreover, this FFQ could also be used to assess dietary habits of Sri Lankans living in other countries, as they practice similar eating patterns to native Sri Lankans. There is no updated nutrient database in the country. Sri Lankan food composition tables were published in 1979, and since then many chemical analysis techniques have changed. Newer processed food items have been introduced into the market. We used the USDA food composition tables as the backbone of our nutrient database. This is arguably the most comprehensive, standardized, largest and continuously updated database that has used to develop population-specific food composition tables in other countries [25]. Mixed dishes were not listed in the USDA database, and for such items we followed calculated values from traditional recipes. The recipes vary according to ethnic groups in Sri Lanka and were therefore modified to allow generalization to the whole country.

### Limitations

Coconut oil is the main cooking oil in Sri Lanka [32], however, other types of cooking oils are used in different communities. Our FFQ does not enable us to differentiate the types of cooking oil consumed which may have important health implications for NCDs. However, we used additional questions to obtain details of oil consumption. Another limitation is the lack of data on micronutrients on Sri Lankan mixed dishes, prolonged cooking time and addition of various spicies and herbs which could alter the nutritional values of the raw ingredients [33]. Moreover, dietary surveys and 24 dietary recalls inherit some limitation such as recall bias, interviewer bias and selection bias.

## Conclusion

This study highlights the development of a FFQ and the related nutrient composition database for Sri Lankan adults. Culturally specific dietary tools are central to capturing the role of diet in risk for chronic disease in Sri Lanka. While the reproducibility and validity of this FFQ needs to be determined, an important ongoing program would be the regular updating of the new nutrient database we have also developed.

## Competing interests

The authors declare that they have no competing interests.

## Authors’ contributions

RJ contributed to the data collection, data analysis and drafted the manuscript. NMB, MJS, PK and APH were supervisory team members on the project and contributed to study design, interpretation of data and revision of the manuscript. All authors read and approved the final manuscript.

## Supplementary Material

Additional file 1 **Figure S1. **Example of a food photograph (200gms of rice). Click here for file

## References

[B1] Somatunga LC NCD Risk factor survey in Sri Lanka (STEP Survey) 2004 World Health Organisation, Geneva www.who.int/chp/steps/SriLankaSTEPSReport2003.pdf [Accessed April 2012]

[B2] Stamler J Assessing diets to improve world health: nutritional research on disease causation in populations Am J Clin Nutr 1994 59 1 146S 8279413 10.1093/ajcn/59.1.146S

[B3] Lee RD Nieman DC Nutritional Assessments 2002 3 Science Engineering, McGraw-Hill

[B4] Willett W Nutrition Epidermiology 1998 2 Oxford University Press, New York

[B5] Perera WDA Jayasekara PM Thaha SZ Tables of food composition for use in Sri Lanka 1979 Colombo

[B6] FAO FAO-Nutrition Country Profiles 1999 Food and Agriculture organization of the United Nations, Rome

[B7] Popkin BM HS Kim S The Nutritional Transition and Diet-Related Chronic Diseases in Asia: Implications for Prevention 2001 International Food Policy Research Institute FCND Discussion Paper, Washington, DC 105

[B8] Shekar M Somanathan A Du L Bank W Malnutrition in Sri Lanka: Scale, Scope, Causes, and Potential Response Human Development Unit, South Asia Region 2007

[B9] Katulanda P Prevalence and projections of diabetes and pre-diabetes in adults in Sri Lanka—Sri Lanka diabetes, cardiovascular study (SLDCS) Diabet Med 2008 25 9 1062 1069 10.1111/j.1464-5491.2008.02523.x 19183311

[B10] Wijewardene K Prevalence of hypertension, diabetes and obesity: baseline findings of a population based survey in four provinces in Sri Lanka Ceylon Med J 2005 50 2 62 70 16114771 10.4038/cmj.v50i2.1571

[B11] Katulanda P Prevalence of overweight and obesity in Sri Lankan adults Obes Rev 2010 10.1111/j.1467-789X.2010.00746.x 20406417

[B12] Katulanda P Ranasinghe P Jayawardena R Metabolic syndrome among Sri Lankan adults: prevalence, patterns and correlates Diabetology & Metabolic Syndrome 2012 4 24 10.1186/1758-5996-4-24 22650800 PMC3407762

[B13] Samaranayake UMM Food Base Dietary Guidelines for Sri Lanka. 2011, Colombo 2011 Nutrition Devision, Ministry of Healthcare and Nutrition, Sri Lanka

[B14] Jayawardena R Byrne NM Soares MJ Katulanda P Hills AP Consumption of Sri Lankan adults: an appraisal of serving characteristics Public Health Nutr 2012 First View 1 6 22784794 10.1017/S1368980012003011 PMC10271271

[B15] Nelson M Atkinson M Meyer J A Photographic Atlas of Food Portion Sizes 1997 MAFF publications, UK

[B16] Shahar S Yusoff NAM Safii NS Ghazau R Ahmad R Atlas of Food Exchanges & Portion Sizes 2009 MDC Publishers, Kuala Lampur

[B17] USDA Foods List. In National Nutrient Database for Standard Reference, 24 2012 National Agricultural Library, editor

[B18] Gopalan C Ramshastri BV Balasubramanian SC Nutritive value of Indian foods 1989 National Institute of Nutrition, Hyderabad

[B19] Welch AA Unwin ID Buss DH Paul AA Southgate DAT McCance and Widdowson’s The Composition of Foods 1995 5 Royal Society of Chemistry, Cambridge

[B20] Dissanayake C Ceylon Cookery 2010 9 Stamford Lake, Sri Lanka (pvt) Ltd

[B21] Hodge A Patterson AJ Brown WJ Ireland P Giles G The Anti Cancer Council of Victoria FFQ: relative validity of nutrient intakes compared with weighed food records in young to middle-aged women in a study of iron supplementation Aust N Z J Public Health 2000 24 6 576 586 10.1111/j.1467-842X.2000.tb00520.x 11215004

[B22] Kobayashi T Development of a food frequency questionnaire to estimate habitual dietary intake in Japanese children Nutrition Journal 2010 9 1 17 10.1186/1475-2891-9-17 20380735 PMC2873262

[B23] Ireland P Jolley D Giles G O'Dea K Powles J Rutishauser I Wahlqvist ML Williams J Development of the Melbourne FFQ: a food frequency questionnaire for use in an Australian prospective study involving an ethnically diverse cohort Asia Pacific J Clin Nutr 1994 3 19 31 24351203

[B24] Bharathi A Development of food frequency questionnaires and a nutrient database for the prospective Urban and Rural Epidemiological (PURE) pilot study in South India: methodological issues Asia Pacific Journal of Clinical Nutrition 2008 17 1 178 185 18364343

[B25] Dehghan M Development of a semi-quantitative food frequency questionnaire for use in United Arab Emirates and Kuwait based on local foods Nutr J 2005 4 1 18 10.1186/1475-2891-4-18 15921524 PMC1166573

[B26] Rahav G Suppl 2006 41 1 i47 155 10.1093/alcalc/agl075 17030503

[B27] Frobisher C Maxwell SM The estimation of food portion sizes: a comparison between using descriptions of portion sizes and a photographic food atlas by children and adults J Hum Nutr Diet 2003 16 3 181 188 10.1046/j.1365-277X.2003.00434.x 12753111

[B28] Huybregts L Validity of photographs for food portion estimation in a rural West African setting Public Health Nutr 2008 11 06 581 587 17686204 10.1017/S1368980007000870

[B29] Caster WO Systematic estimation of food intakes from food frequency data Nutr Res 1986 6 4 469 472 10.1016/S0271-5317(86)80189-0

[B30] Cancer Council Victoria. Dietary questionnaires 2010 Available from: http://www.cancervic.org.au/about-our-research/epidemiology/nutritional_assessment_services. [Accessed May 2012]

[B31] Ocké MC The Dutch EPIC food frequency questionnaire. I. Description of the questionnaire, and relative validity and reproducibility for food groups Int J Epidemiol 1997 26 suppl 1 S37 9126532 10.1093/ije/26.suppl_1.s37

[B32] Amarasiri WA Dissanayake AS Coconut fats Ceylon Med J 2006 51 2 47 51 17180807 10.4038/cmj.v51i2.1351

[B33] Khokhar SRM Swan G Carotenoid and retinol composition of South Asian foods commonly consumed in the UK J Food Compos Anal 2012 25 2 166 172 10.1016/j.jfca.2011.11.005

