# Supplementary figures and images for: Development of a food frequency questionnaire for Sri Lankan adults
Source: Nutr J. 2012 Aug 31;11:63. doi: 10.1186/1475-2891-11-63 (PMC3496639; doi:10.1186/1475-2891-11-63)

## Supplementary file 1

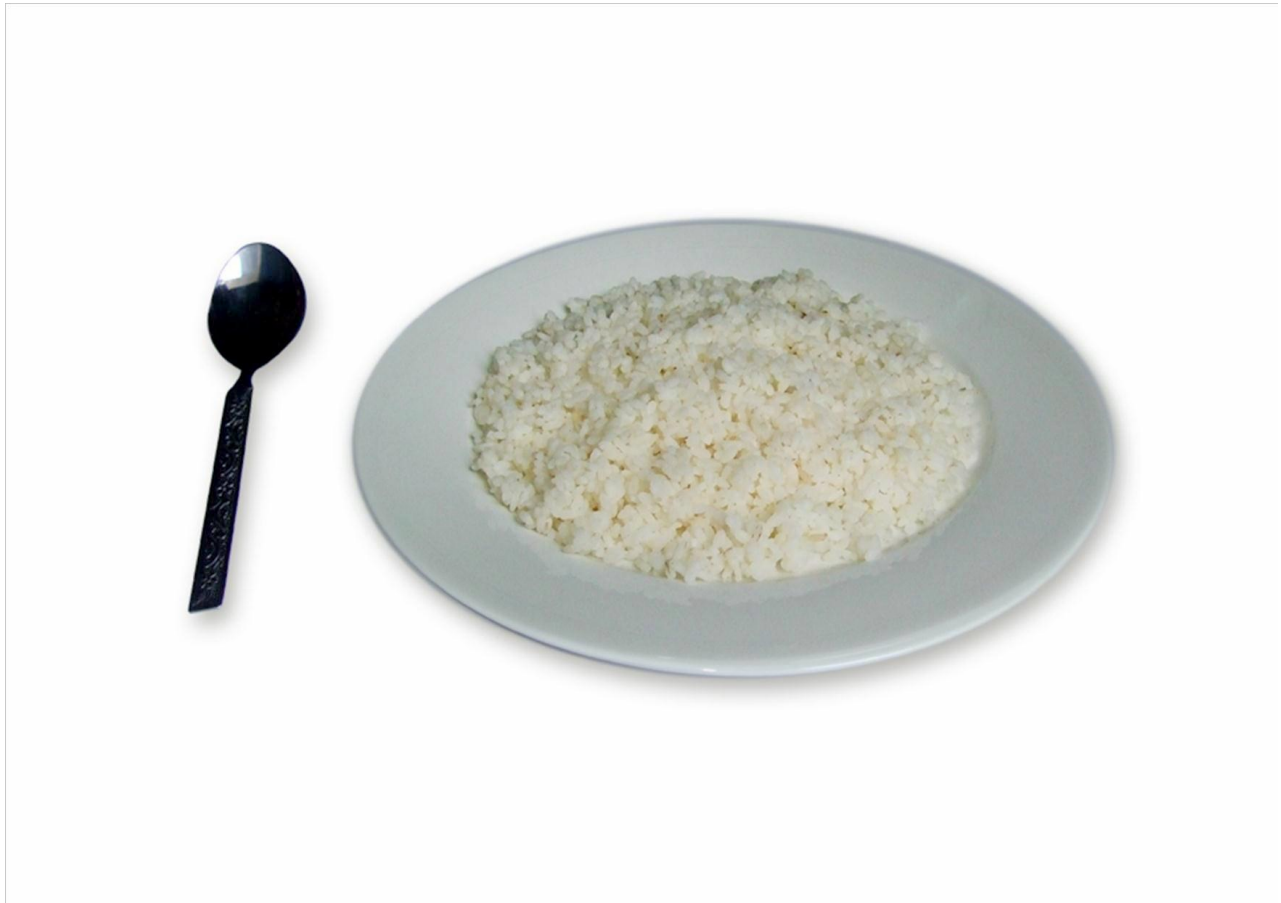

**Figure 1:** Example of a food photograph (200gms of rice)

Supplement: Additional file 1 — Figure S1. Example of a food photograph (200gms of rice). [file 1475-2891-11-63-S1.pdf]
